# Origin of eukaryotes from within archaea, archaeal eukaryome and bursts of gene gain: eukaryogenesis just made easier?

**DOI:** 10.1098/rstb.2014.0333

**Published:** 2015-09-26

**Authors:** Eugene V. Koonin

**Affiliations:** National Center for Biotechnology Information, National Library of Medicine, National Institutes of Health, Bethesda, MD 20894, USA

**Keywords:** endosymbiosis, phagocytosis, cytoskeleton, horizontal gene transfer, archaea

## Abstract

The origin of eukaryotes is a fundamental, forbidding evolutionary puzzle. Comparative genomic analysis clearly shows that the last eukaryotic common ancestor (LECA) possessed most of the signature complex features of modern eukaryotic cells, in particular the mitochondria, the endomembrane system including the nucleus, an advanced cytoskeleton and the ubiquitin network. Numerous duplications of ancestral genes, e.g. DNA polymerases, RNA polymerases and proteasome subunits, also can be traced back to the LECA. Thus, the LECA was not a primitive organism and its emergence must have resulted from extensive evolution towards cellular complexity. However, the scenario of eukaryogenesis, and in particular the relationship between endosymbiosis and the origin of eukaryotes, is far from being clear. Four recent developments provide new clues to the likely routes of eukaryogenesis. First, evolutionary reconstructions suggest complex ancestors for most of the major groups of archaea, with the subsequent evolution dominated by gene loss. Second, homologues of signature eukaryotic proteins, such as actin and tubulin that form the core of the cytoskeleton or the ubiquitin system, have been detected in diverse archaea. The discovery of this ‘dispersed eukaryome’ implies that the archaeal ancestor of eukaryotes was a complex cell that might have been capable of a primitive form of phagocytosis and thus conducive to endosymbiont capture. Third, phylogenomic analyses converge on the origin of most eukaryotic genes of archaeal descent from within the archaeal evolutionary tree, specifically, the TACK superphylum. Fourth, evidence has been presented that the origin of the major archaeal phyla involved massive acquisition of bacterial genes. Taken together, these findings make the symbiogenetic scenario for the origin of eukaryotes considerably more plausible and the origin of the organizational complexity of eukaryotic cells more readily explainable than they appeared until recently.

## 1. Introduction

The origin of eukaryotes is one of the hardest and most intriguing problems in the study of the evolution of life, and arguably, in the whole of biology. Compared to archaea and bacteria (collectively, prokaryotes), eukaryotic cells are three to four orders of magnitude larger in volume and display a qualitatively higher level of complexity of intracellular organization [1–3]. Unlike the great majority of prokaryotes, eukaryotic cells possess an extended system of intracellular membranes that includes the eponymous eukaryotic organelle, the nucleus, and fully compartmentalizes the intracellular space. In eukaryotic cells, proteins, nucleic acids and small molecules are distributed by specific trafficking mechanisms rather than by free diffusion as is largely the case in bacteria and archaea [4,5]. Thus, eukaryotic cells function on different physical principles compared to prokaryotic cells, which is directly due to their (comparatively) enormous size.

The gulf between the cellular organizations of eukaryotes and prokaryotes is all the more striking because no intermediates have been found. Comparative analysis of eukaryotic cells and genomes confidently maps highly advanced functional systems and macromolecular complexes to the last eukaryotic common ancestor (LECA). The actin and tubulin cytoskeletons, the nuclear pore, the spliceosome, the proteasome and the ubiquitin signalling system are only a few of the striking examples of the organizational complexity that seems to be a ‘birthright’ of eukaryotic cells [6–10]. The formidable problem that these fundamental complex features present to evolutionary biologists makes Darwin's famous account of the evolution of the eye look like a simple, straightforward case. Indeed, so intimidating is the challenge of eukaryogenesis that the infamous notion of ‘irreducible complexity’ has sneaked into serious scientific debate [11], albeit followed by a swift refutation [12].

Molecular phylogenetics and phylogenomics revealed fundamental aspects of the origin of eukaryotes. The ‘standard model’ of molecular evolution, derived primarily from the classic phylogenetic analysis of 16S RNA by Woese and co-workers and supported by subsequent phylogenetic analyses of universal genes, identifies eukaryotes as the sister group of archaea, to the exclusion of bacteria [13–16]. Within the eukaryotic part of the tree, early phylogenetic studies have placed into the root position several groups of unicellular organisms, primarily parasites, that unlike the majority of eukaryotes, lack mitochondria. These organisms have been construed as ‘archezoa’, i.e. the primary amitochondrial eukaryotes that were thought to have hosted the proto-mitochondrial endosymbiont [17–20].

However, advances of comparative genomics jointly with discoveries of cell biology have put the archezoan scenario of eukaryogenesis into serious doubt. First, it has been shown that all the purported archezoa possess organelles, such as hydrogenosomes and mitosomes, that appeared to be derivatives of the mitochondria. These mitochondria-like organelles typically lack genomes but contain proteins encoded by genes of apparent bacterial origin that encode homologous mitochondrial proteins in other eukaryotes [21,22]. Combined, the structural and phylogenetic observations leave no reasonable doubt that hydrogenosomes and mitosomes indeed evolved from the mitochondria. Accordingly, no primary amitochondrial eukaryotes are currently known, suggesting that the primary *α*-proteobacterial endosymbiosis antedated the LECA [22–24]. Compatible with this conclusion, subsequent, refined phylogenetic studies have placed the former ‘archezoa’ within different groups of eukaryotes indicating that their initial position at the root was an artefact caused by their fast evolution, most probably causally linked to the parasitic lifestyle [25–27]. These parallel developments left the archezoan scenario without concrete support but have not altogether eliminated its attractiveness. An adjustment to the archezoan scenario simply posited that the archezoa was an extinct group that had been driven out of existence by the more efficient mitochondrial eukaryotes [28,29]. A concept predicated on an extinct group of organisms that is unlikely to have left behind any fossils and is refractory to evolutionary reconstruction due to the presence of mitochondria (or vestiges thereof) in all eukaryotes is quite difficult to refute but can hardly get much traction without any concrete evidence of the existence of archezoa.

The radical alternative to the elusive archezoa is offered by symbiogenetic scenarios of eukaryogenesis according to which archezoa, i.e. primary amitochondrial eukaryotes, have never existed, and the eukaryotic cell is the product of a symbiosis between two prokaryotes [1,2,12,23,30,31]. Comparative genomic analysis clearly demonstrates that eukaryotes possess two distinct sets of genes, one of which shows phylogenetic affinity with homologues from archaea, whereas the other one includes genes affiliated with bacterial homologues (apart from these two classes, there are many eukaryotic genes of uncertain provenance). The eukaryotic genes of apparent archaeal descent encode, primarily, proteins involved in information processing (translation, transcription, replication, repair), whereas the genes of inferred bacterial origin encode mostly proteins with ‘operational’ functions such as metabolic enzymes, components of membranes and other cellular structures and others [32–35]. Notably, altogether, the number of eukaryotic protein-coding genes of bacterial origin exceeds the number of ‘archaeal’ proteins about threefold. Thus, although many highly conserved, universal genes of eukaryotes indeed appear to be of archaeal origin, the archaeo-eukaryotic affinity certainly does not tell the entire story of eukaryogenesis, not even most of that story if judged by the proportions of genes of apparent archaeal and bacterial descent.

Although several symbiogenetic scenarios that differ in terms of the proposed partners and even the number of symbiotic events involved have been proposed, the simplest, parsimonious one that accounts for both the ancestral presence of mitochondria in eukaryotes and the hybrid composition of the eukaryotic gene complement involves engulfment of an *α*-proteobacterium by an archaeon [12,30,33,36]. Under this scenario, a chain of events has been proposed that leads from the endosymbiosis to the emergence of eukaryotic innovations such as the endomembrane system, including the nucleus and the cytoskeleton [36,37]. Subsequently, argument has been developed that the energy demand of a eukaryotic cell that is orders of magnitude higher than that of a typical prokaryotic cell cannot be met by means other than utilization of multiple ‘power stations’ such as the mitochondria [1,2,31].

A major problem faced by this scenario (and symbiogenetic scenarios in general) is the mechanistic difficulty of the engulfment of one prokaryotic cell by another [20,28,29,38]. Although bacterial endosymbionts of certain proteobacteria have been described [39,40], such a relationship appears to be a rarity. By contrast, in many unicellular eukaryotes, such as amoeba, engulfment of bacterial cells is routine due to the phagotrophic lifestyle of these organisms [20]. The apparent absence of phagocytosis in archaea and bacteria prompted the reasoning that the host of the proto-mitochondrial endosymbiont was a primitive phagotrophic eukaryote, which implies the presence of an advanced endomembrane system and cytoskeleton [20,28,29,38]. Thus, argument from cell biology seemed to justify rescuing the archezoan scenario, the lack of positive evidence notwithstanding.

However, comparative analysis of the increasingly diverse collection of archaeal and bacterial genomes has yielded multiple lines of evidence that might change the notion of the implausibility of an archaeo-bacterial endosymbiosis. In this article, I discuss the results of genome evolution reconstructions that imply complex ancestral archaeal forma and the discovery of the dispersed archaeal ‘eukaryome’. The eukaryome consists of multiple genes identified in different archaea that encode key components of the cytoskeleton, the cell division apparatus, the ubiquitin system and other signature eukaryotic cellular systems. A complementary line of recent developments shows that massive acquisition of bacterial genes probably occurred on multiple occasions in the course of the evolution of archaea. Taken together, these findings seem to be making the scenario of archaeo-bacterial symbiosis considerably more plausible than it appeared even recently.

## 2. Burgeoning archaeal diversity, complex archaeal ancestor and origin of eukaryotes from within the archaea

As pointed out above, the ‘standard model’ phylogeny of Woese and co-workers clearly identifies archaea and eukaryotes as sister groups [13–15]. However, an alternative phylogeny inferred from trees of the same 16S rRNAs using a different phylogenetic method and compatible also with the results of ribosome structure comparison appeared shortly after the publication of the three-domain tree of life [41,42]. That alternative topology led to the eocyte hypothesis under which eukaryotes evolved from within the archaea and are a sister group to the ‘eocytes’, i.e. the archaeal phylum that is currently known as Crenarchaeota [41–44]. Support for the eocyte hypothesis has been subsequently reported from comparative analysis of ribosomal protein sequences that did not involve phylogeny reconstruction [45] and from a novel phylogenomic approach [33]. A later, sophisticated phylogenetic analysis of multiple conserved genes that employed a technique eliminating fast-evolving alignment columns and has been reported to minimize the effect of common artefacts of phylogenetic analysis, such as long-branch attraction, has supported the affinity of eukaryotes with Crenarchaeota [46]. The alternative topologies including the standard three-domain phylogeny with archaea and eukaryotes as sister groups have been deemed to result from phylogenetic artefacts that are overcome by this approach.

The eocytes (Crenarchaeota) are not the only group of archaea that has been proposed for the role of the archaeal ancestor of eukaryotes. Evolutionary scenarios based on different versions of metabolic cooperation between the archaeal and bacterial partners of the primary ensodymbiosis, such as the hydrogen hypothesis [30] and the syntrophic hypothesis [47], implied origin of the ‘archaeal’ genes of eukaryotes from euryarchaea, and in particular, methanogens. The methanogen affinity for eukaryotes has been claimed also from some phylogenetic analyses [48,49]. Yet other phylogenetic studies have produced results compatible with the standard model, placing eukaryotes outside the known archaeal diversity [50–53]. Biological considerations, such as the greater role of RNA in a variety of processes in eukaryotic cells (splicing, defence, regulation of gene activity and more), have even led to the idea that eukaryotes were the first cellular life forms [54–56].

Most of the inferences of archaeo-eukaryotic relationship studies mentioned above were based either on phylogenetic analysis of a single, universal gene, such as 16S rRNA, or on concatenated sequences of several universal proteins (e.g. ribosomal proteins), or on non-sequence characters such as gene repertoires (phyletic patterns) and domain architectures of multidomain proteins. Obviously, extensive sequencing of genomes from all three domains of cellular life opens the door for comprehensive phylogenomic analyses. The first such extensive phylogenomic exercise involved analysis of nearly 6000 gene sets from 185 archaeal, bacterial and eukaryotic genomes and employed a supertree approach to combine information from the multiple trees; the results suggested affinity between eukaryotes and the Thermoplasmatales branch of Euryarchaeota, albeit with limited statistical support [34]. *Thermoplasma* or a related, wall-less archaeon also has been proposed as a plausible ancestor of eukaryotes, on the basis of biochemical and cytological considerations, long before the phylogenomic analysis became feasible [57–61].

Another comprehensive phylogenetic analysis of individual eukaryotic genes of apparent archaeal origin has suggested an origin outside of the known archaeal diversity for most of these genes but also identified many genes with a crenarchaeal (eocyte) affinity and a smaller number of genes with a euryarchaeal affinity [62]. In this study, the possibility has been brought up that the discrepancies between the tree topologies for different genes did not necessarily result from phylogenetic artefacts, but instead could reflect joint presence of genes currently identified in different archaea in the genome of the ancestral form that became the host of the proto-mitochondrial endosymbiont.

**Figure 1.**
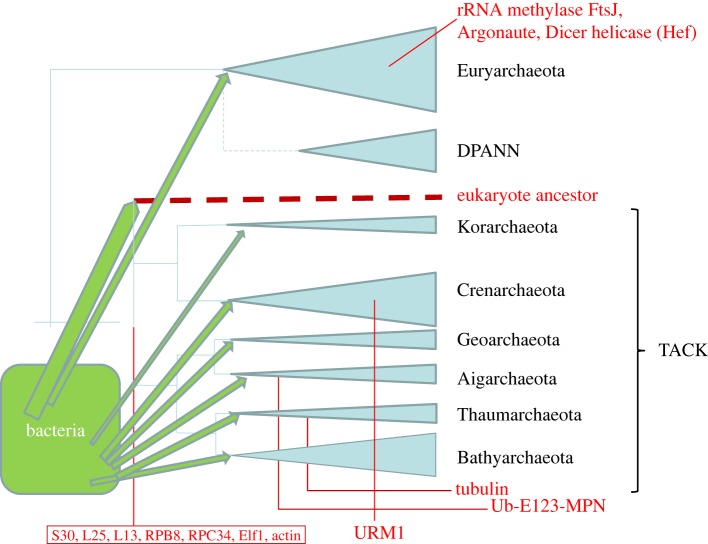
A schematic evolutionary tree of the archaea, the likely origin of eukaryotes and the distribution of eukaryome components. The tree topology is from [73] except that the DPANN branch was moved to the base of the Euryarchaeota according to [75–77]. The size of the triangles very roughly shows the diversity of the respective groups. The hypothetical lineages of eukaryote ancestors are tentatively shown as a deep branch within the TACK superphylum. The inferred origins of some key eukaryotic genes and functional systems [78] are indicated by red lines; S30, L25 and L13 are ribosomal proteins; Ub-E123-MPN denotes the ubiquitin system where E123 are the respective subunits of the ubiquitin ligase and MPN is the deubiquitinase. The green arrows from bacteria denote the gene flow associated with the origin of the major groups of archaea [79]. The thick arrow pointing at the putative ancestor of eukaryotes denotes the massive gene flow from the endosymbiont.

The diversity of the outcomes of phylogenetic analysis, with the origin of eukaryotes scattered around the archaeal diversity, has led to considerable frustration and suggested that a ‘phylogenomic impasse’ has been reached, owing to the inadequacy of the available phylogenetic methods for disambiguating deep relationships [63]. However, the landscape of archaeal phylogenomics started to radically change when genome analysis of several poorly characterized archaea suggested the existence of several new phyla, in particular Korarchaeota [64] and Thaumarchaeota, the latter encompassing mesophilic archaea previously included within the Crenarchaeota [65]. Subsequently, it has been shown that Thaumarchaeota are a widespread microbial group of major geochemical importance that includes, in particular, the key ammonia oxidizers in marine and soil habitats [66–68]. For Korarchaeota, there is still a single complete genome but metagenomic studies suggest substantial diversity in various marine and terrestrial habitats [69,70]. Genome analysis of the uncultivated archaeon *Candidatus Caldiarchaeum subterraneum* has suggested yet another archaeal phylum, dubbed Aigarchaeota [71]. The latest, extensive metagenomic and single-cell genomics studies have led to a veritable ‘bonanza’ of putative new archaeal phyla [67,72–74] (figure 1).

Independent phylogenomic analyses of multiple conserved genes consistently support a deeply rooted archaeal ‘TACK’ superphylum that originally encompassed Thaumarchaeota, Aigarchaeota, Crenarchaeota and Korarchaeota [80–84], but according to the latest comprehensive phylogenetic study, additionally contains two novel phyla, Bathyarchaeota and Geoarchaeota (however, a subsequent re-analysis has suggested inclusion of Geoarchaeota into Crenarchaeota, thus denying this group the status of a new phylum (figure 1)). This new phylogeny also includes another putative superphylum designated DPANN that combines Nanoarchaeota and other archaeal groups with small genomes.

A recent detailed phylogenomic study that included an expanded set of nearly universal phylogenetic markers and improved phylogenetic methods has confidently placed the root of the archaeal tree between the Euryarchaeota, including Nanoarchaeota and other fast-evolving groups, and the rest of the archaeal phyla that comprise the TACK superphylum, or proposed new kingdom Proteoarchaeota [75,76] (figure 1). However, other recent phylogenomic analysis using different techniques and datasets variously place the root between the DPANN superphylum and the rest of the Archaea [85] or within the Euryarcheota [86,87]. Thus, the archaeal root position remains an open problem. Given that in order to establish the root position for the Archaea the use of a representative sample of bacterial homologues as an outgroup is essential, attempts to solve this problem involve the deepest relationships between cellular life forms for which detection of an unequivocal signal is inherently difficult.

The monophyly of the TACK superphylum is further buttressed by the reconstruction of the evolution of the archaeal gene repertoire that revealed probably massive gene gain at the base of the TACK branch (figure 1) [84]. This reconstruction, which extended previous analyses [88,89], reveals a remarkable trend in the evolution of archaea that is likely to reflect a general pattern of genome evolution. This general tendency consists in the dominance of genome reduction at the most common course of evolution that is punctuated with episodes of explosive genome expansion [90]. These reconstructions indicate that each of the major archaeal lineages underwent some degree of genome reduction in the course of its evolution and that the gene complement of the last archaeal common ancestor (LACA) was at least as complex as that of most of the extant archaea. Reductive evolution associated with genome streamlining appears to be an extremely general evolutionary phenomenon characteristic of successful groups that reach large effective population sizes and evolve under strong selective pressure [90]. However, evolution of the archaea might be specifically conducive to genome reduction as part of adaptation to high stress conditions [54,91].

Identification of new archaeal phyla and the putative TACK superphylum stimulated further phylogenomic effort aimed at the elucidation of the archaeal ancestry of eukaryotes. Two independent, detailed phylogenetic analyses of rRNA and universal protein-coding genes employing state-of-the-art phylogenetic methods have shown significant support for the monophyly of eukaryotes with the TACK superphylum but not with any specific lineage within the TACK [81,92,93]. An alternative analysis has placed eukaryotes within the TACK superphylum, as a sister group to Thaumarchaeota [94]. The controversy over the phylogenetic position of eukaryotes, or more precisely, universal eukaryotic genes encoding translation system components, vis-a-vis archaea has not been put to rest by these analyses. Thus, a phylogenomic analysis that focused on the archaeal ‘dark matter’, i.e. sequences from numerous uncultivated organisms, has supported the standard model topology, i.e. eukaryotes outside the archaea [72]. However, a re-analysis that employed a better fitting phylogenetic model and excluded some eukaryotic genes of mitochondrial and chloroplast origin that appeared to have crept into the dark matter study has once again recovered the eukaryote–TACK affinity [85].

Importantly, an evolutionary affinity between eukaryotes and the TACK superphylum is compatible with a series of findings that are independent of phylogenetic methodology. It has been shown that several ancestral genes are shared exclusively by eukaryotes and archaea of the TACK superphylum, in contrast to a smaller number of such shared derived characters between eukaryotes and Euryarchaeota (figure 1) [78]. Notably, the shared derived characters of eukaryotes and the TACK superphylum include several components of the core information processing system, including three ribosomal proteins [83], the RNA polymerase subunits RPB8 [95] and RPC34 [96] and the transcription factor Elf1 [97]. Genes in this category are relatively rarely transferred horizontally, so the shared ones are likely to come from the common ancestor of the respective groups, in this case, eukaryotes and the TACK superphylum archaea.

Taking together all the relevant lines of evidence, it appears that, although claiming a definitive solution could be premature, a consensus is shaping up on the specific origin of the archaeal heritage of eukaryotes. Most of the genes that eukaryotes inherited from archaea appear to originate from the TACK superphylum, although there are interesting exceptions to this pattern as discussed below (figure 1). Thus, the results of increasingly robust phylogenomic analyses appear to be best compatible with a model of two primary domains of cellular life, Archaea and Bacteria, with eukaryotes emerging from within the Archaea, as opposed to the standard three-domain model [93]. This conclusion does not rule out the possibility that the eukaryotic ancestor that evolved from a common root with or from within the TACK and eventually hosted the proto-mitochondrial endosymbiont was an ‘archezoan’ but appears to fit more smoothly into a scenario of the host being a bona fide archaeon.

## 3. Massive gene gain: apparent common denominator in the origin of new archaeal phyla

As pointed out above, reconstructions of the evolution of the archaeal gene complement imply episodic gene gain, conceivably associated with the emergence of major groups, followed by gradual gene loss leading to genome streamlining in multiple lineages [84,90]. Recently, this view of archaeal evolution has received strong support from focused studies of acquisition of bacterial genes by archaea. Ever since the first genomes of mesophilic archaea were reported, it has become clear that these organisms contain numerous genes of apparent bacterial origin, many more than archaeal thermophiles [98–100]. Subsequent phylogenomic study of Halobacteria have revealed a striking pattern: over 1000 bacterial genes apparently have been acquired by a methanogenic ancestor of Halobacteria and recruited for the characteristic halobacterial metabolic pathways [101]. Thus, this massive capture of bacterial genes seems to have led to the emergence of a major group of archaea. Comprehensive phylogenomic analysis of all available archaeal and bacterial genomes has expanded these observations by showing that capture of multiple bacterial genes is characteristic of 13 major groups of archaea [79]. Moreover, topologies of the phylogenetic trees appear to be best compatible with massive acquisition of the bacterial genes at the base of each archaeal branch as opposed to piecemeal acquisition along the branch (figure 1).

These findings have obvious and striking implications for the origin of eukaryotes. Acquisitions of numerous bacterial genes that amount to genomic chimaerism and lead to substantial remolding of cell physiology and emergence of groups with new lifestyles appears to be a recurrent rather than unique event in evolution, at least in archaea. Could it be that most if not all major groups of archaea emerged from botched endosymbiotic events? Should that be the case, eukaryogenesis only differs in that the endosymbiont survived, retaining part of its physical and genetic identity.

## 4. The scattered archaeal eukaryome

The comparative genomic observations discussed above seem to increase the plausibility of an archaeal host for the mitochondrial endosymbiont and further indicate that evolutionary events leading to massive acquisition of bacterial genes were relatively common in archaeal evolution. Yet, the main obstacle faced by the symbiogenetic scenarios of eukaryogenesis, namely the mechanistic difficulty of engulfment of one prokaryotic cell by another, has remained as formidable as ever. As long as the chance of an archaeon engulfing a bacterium is considered to be vanishingly low, the symbiogenetic scenarios can be dismissed as unrealistic [28,29].

However, the latest findings of comparative genomics cast this thorny issue in a different light. It has become clear that, apart from the core of the universal information processing systems, probably archaeal ancestors of signature eukaryotic genes and entire functional systems that have to do with the intracellular architecture are often found in diverse groups of archaea. We denote this set of genes the ‘eukaryome’ to emphasize their specific importance for the biology of eukaryotic cells [78].

Unexpectedly, for a substantial number of ancestral eukaryotic genes, homologues have been detected in only one group of archaea. These lineage-specific ancestral genes are scattered across the entire archaeal domain but are most common in the TACK superphylum and in particular in *Ca. Caldiarchaeum subterraneum*, so far the only representative of Aigarchaeota with a complete genome [78]. Below I discuss the most striking cases of the dispersal of the eukaryome components among archaea.

### (a) The cytoskeleton

The indispensable structural framework of all eukaryotic cells is the advanced, elaborate cytoskeleton that consists of two major types of elements, namely actin-based filaments and tubulin-based microtubules [102,103]. The cytoskeleton is central to the discussion of the origin of eukaryotes, in particular because actin filaments play the key role in phagocytosis, the process that is considered critical for the engulfment of the proto-mitochondrial endosymbiont by its host, whatever the exact nature of the latter [38]. Until recently, bacteria and archaea have been thought to encode only distant homologues of actin and tubulin, the proteins of the MreB/FtsA and FtsZ families, respectively, that perform essential functions in the septation of bacterial and some archaeal cells [104,105]. The sequence similarity between the bacterial and archaeal proteins of these families and the eukaryotic cytoskeleton components is rather low, so that homology has been considered firmly established only through structural comparisons [106–109].

Recently, analysis of the expanding archaeal genome collection has changed this perspective. Proteins with high sequence similarity and unambiguous phylogenetic affinity to eukaryotic actins have been discovered in the crenarchaeal order of Thermoproteales, *Korarchaeum* and *Ca. Caldiarchaeum subterraneum*, with the implication that actin was already present in the last common ancestor of the TACK superphylum [110,111]. Following these findings of comparative genomics, it has been shown that archaeal actin homologues, named crenactins, actually form helical filaments resembling typical eukaryotic actin filaments [112–114].

Sequence analysis of the crenactins has indicated that these proteins contained several insert that are present in eukaryotic actin-related proteins (ARPs) but not in actins themselves [110]. Once two crenactin structures have been solved, these inserts have been shown to form extended loops [115,116]. The corresponding loops in the ARPs are required for the formation of branched filaments which are involved in phagocytosis [117]. This similarity led to the proposition that crenactin filaments could endow some members of the TACK superphylum with at least a rudimentary phagocytic capacity [110].

Highly conserved orthologues of tubulins, named artubulins, so far have been discovered only in the genomes of several ammonium-oxidizing Thaumarchaeota of the genera *Nitrosoarchaeum* and *Nitrosotenius* [118,119]. Although in this case horizontal gene transfer (HGT) from eukaryotes to archaea could not be technically ruled out, phylogenetic analysis results appear to be best compatible with an ancestral status of the artubulins with respect to the eukaryotic tubulins [118]. The structures and functions of the artubulins remain to be characterized but it appears highly likely that they form a microtubule-type cytoskeleton.

Thus, in a remarkable departure from recent common beliefs, both major forms of the eukaryotic cytoskeleton seem to belong within the archaeal heritage of eukaryotes. However, the apparent ancestral forms have been detected in widely different groups of extant archaea.

### (b) Cell division and membrane remodelling systems

Cell division obviously is central to all cellular life forms. Nevertheless, the cell division mechanisms substantially differ between bacteria and at least some archaea, on the one hand, and eukaryotes, on the other hand. In bacteria, division is coupled to chromosome replication, with the progeny DNA molecules being pumped into the daughter cells concomitant with replication. Division is then completed by septation, with the formation of the septum driven by the Z-ring that consists of the FtsZ protein, a GTPase that is a distant homologue of eukaryotic tubulins [120,121]. In addition to nearly all bacteria, the FtsZ-centred division machinery is encoded in the genomes of most of the Euryarchaeota and Thaumarchaeota as well as *Korarchaeum*, with the implication that the division mechanisms of these archaea are similar to the bacterial one [111].

Unexpectedly, a distinct division system homologous to the eukaryotic ESCRT-III membrane remodelling complex has been discovered in the crenarchaeon *Sulfolobus acidocaldarius* [122–125] and subsequently identified with comparative genomic methods in all members of two of the three crenarchaeal orders, *Sulfolobales* and *Desulfurococcales*, as well as some Thaumarchaeota and Euryarchaeota [111]. Subsequently, it has been shown that the ESCRT-III-like complex is the primary cell division system in the thaumarchaeon *Nitrosopumilus maritimus* [126]. Furthermore, one of the ESCRT-III protein homologues, CdvA of *Sulfolobus acidocaldarius*, has been shown to form helical filaments that mediate membrane scission during cell division [127]. This finding reveals the distinct form of cytoskeleton that is required for division in ESCRT-III-encoding archaea.

The presence of the FtsZ-based and ESCRT-III-like division systems in a broad variety of diverse archaea implies that both machineries were present in the LACA, with subsequent differential losses in multiple lineages. The Crenarchaeota in the order Thermoproteales lack both of these division systems and thus must employ a distinct third one that most probably relies on the crenactin cytoskeleton [111,113]. Given that the origin of crenactin can be mapped to the base of the TACK superphylum (figure 1 and see above), one comes to the striking conclusion that the common ancestor of the TACK most probably possessed all three cell division systems that are scattered among the extant archaea (although the likelihood of this conclusion depends on the position of the archaeal root). Discovery of additional distinct variants of the cell division apparatus in archaea appears plausible. At least one available archaeal genome, that of *Picrophilus torridus* (order Thermoplasmatales), lacks all three division machineries discussed above and hence is expected to employ a novel mechanism [111].

### (c) The ubiquitin signalling system

The ubiquitin (Ub) system is the central signalling and regulatory network of the eukaryotic cell. This extremely complex machinery regulates protein degradation, topogenesis and function in all eukaryotes through modification of proteins by conjugation with various forms of (poly)Ub and its paralogues [128–130]. For many years, the Ub system had been considered a key eukaryotic innovation that seemed to have evolved by the exaptation route, i.e. recruitment of prokaryotic enzymes involved in thiamine and molibdopterin coenzyme biosynthesis for a novel function [131,132]. Subsequently, it has been shown that, with the participation of a homologue of the E1 subunit of eukaryotic Ub ligases, some of the archaeal Ub homologues are conjugated with proteins and target them for degradation [133–135]. However, the proteins involved in these processes are distant homologues of Ub and E1, so this discovery did not necessarily shed light directly on the origin of the eukaryotic Ub systems; in particular, the origin of the E2 and E3 subunits of the Ub ligases remained elusive.

Analysis of the genome of *Ca. Caldiarchaeum subterraneum*, the founding member of the putative phylum Aigarchaeota, has transformed the entire story of the origin of the Ub system by revealing a predicted operon encoding a Ub-like protein and homologs of all three Ub ligase subunits along with a key deubiquitinating enzyme [71]. Operons with a similar organization of Ub-related genes have been identified also in several bacteria suggestive of horizontal dissemination of these operons among prokaryotes [78]. In contrast to the distant homologues of Ub system components that have been detected previously in other archaea, the homologues from *Ca. Caldiarchaeum subterraneum* show high sequence similarity to eukaryotic counterparts, and phylogenetic analysis of the E1 subunit of Ub ligase and the deubuiqitinating enzyme MPN (the two largest and most conserved proteins in the Ub system that accordingly are conducive to phylogenetic analysis) place *Ca. Caldiarchaeum subterraneum* in the midst of eukaryotes [78]. Subsequent comparative genomic analysis has led to the identification of similar predicted operons in multiple genomes of Aigarchaeota that have been assembled from metagenomic sequences [136]. The possibility of acquisition of the Ub system by archaea from eukaryotes via HGT can be ruled out given the operonic organization of the archaeal genes. Thus, to date, Aigarchaeota encode the best candidate for the ancestor of the eukaryotic Ub system.

Other homologues of the Ub system components are scattered among archaea [136,137]. In particular, archaeal orthologues of the distant Ub homologue Urm1, which is conserved in all eukaryotes and performs a dual function as a sulfur carrier in tRNA thiolation and in protein modification [138,139], are detectable only among the members of the crenarchaeal order Sulfolobales [137] (figure 1). Thus, within the broadly defined Ub system, at least two distinct archaeal ancestors of essential eukaryotic functional modules have been detected.

### (d) The RNA interference system (RNAi)

The RNAi is a hallmark eukaryotic functional system that is involved in the defence against viruses and transposons and in multiple pathways of gene expression regulation [140–142]. Phylogenomic reconstructions suggest that LECA already possessed a diversified RNAi system [140–143]. The diverse RNAi mechanisms are centred around two key protein families, the Dicers and the Argonautes. The Dicers combine helicase and RNAse activities and are primarily responsible for the processing of small interfering (si) RNAs and microRNAs [144–147]. The Argonautes are nucleases of the RNAse H superfamily, some of which directly attack the RNA targets of RNAi (slicers) whereas others bind microRNAs and guide it to the target with cleaving of the latter [145,148,149].

The Dicers are signature eukaryotic proteins that have no direct counterparts in bacteria or archaea and encompass a fusion of a helicase domain and two RNAse III domains that are unique to eukaryotes. The dsRNA-specific RNAse III is nearly ubiquitous in bacteria and apparently has been acquired by some mesophilic Euryarchaeota via HGT [150]. By contrast, the helicase domain of Dicer appears to have evolved from the euryarchaeal helicase–nuclease Hef that is involved in DNA replication and repair [141,151].

In contrast to Dicers, the Argonautes have numerous homologues in bacteria and archaea, primarily Euryarchaeota, and phylogenetic analysis clearly points to a euryarchaeal origin of the eukaryotic Argonaute family [152,153]. Comparative genomic analysis of the gene neighbourhoods of the archaeal and bacterial Argonautes has led to the hypothesis that these proteins are involved in RNA- or DNA-dependent defence against foreign nucleic acids, similar to their eukaryotic homologues [152]. Subsequently, the defence function of Argonautes in bacteria was shown through the demonstration that these proteins employ RNA or DNA guide molecules to target and cleave foreign DNA [154–156].

A third key component of the eukaryotic RNAi is an RNA-dependent RNA polymerase (lost in some eukaryotic lineages including vertebrates) that serves as an amplifier of siRNAs. Homologues and possible ancestors of this polymerase have been identified in some bacteriophages where they are most likely to function in transcription, as DNA-dependent RNA polymerases [141,157].

Thus, the RNAi system, a signature eukaryotic defence and regulatory network, appears to have evolved from the archaeal Argonaute-centred defence machinery through the accretion of additional components. The ancestral archaeal system remains to be thoroughly characterized, and it cannot be ruled out that functional analogues and possibly even direct homologues of Dicers will eventually be discovered in some archaea.

## 5. The archaeal ancestor of eukaryotes: a complex, ancient group within the TACK superphylum with a prototype phagocytic ability?

Arguably, the greatest difficulty faced by the endosymbiotic scenarios of eukaryogenesis is the apparent implausibility (or at least extreme rarity) of the engulfment of one prokaryotic cell by another. The recent advances of comparative genomics, complemented by the progress in the cell biology of archaea, seem to be closing this gap. Combined with the quantitative findings of genome evolution reconstructions on extensive differential gene loss in most archaeal lineages, the discovery of the ‘dispersed’ archaeal eukaryome implies a highly complex archaeal ancestor of eukaryotes [78,86]. Conceivably, this ancestral form possessed advanced cellular organization and certain ‘eukaryote-like’ functional capacities provided by the ancestral versions of various eukaryotic functional systems that are represented in different lineages of extant archaea (figure 1). The critical point is that the hypothetical eukaryotic ancestor probably possessed a cytoskeleton that consisted of both actin filaments and tubulin microtubules and could provide for a primitive phagocytic capacity [82,110]. Furthermore, the likely presence of multiple cell division systems, such as the FtsZ-based machinery and ESCRT-III [111], in the archaeal ancestor of eukaryotes implies that one of these, perhaps the latter, was involved in processes distinct from division proper, such as membrane remodelling, that could contribute to phagocytosis. Indeed, eukaryotic ESCRT complexes are implicated in phagocytosis-related processes, in particular autophagy [158].

Most probably, the archaeal ancestor of eukaryotes was a wall-less mesophile that coexisted with diverse bacteria, so even a limited capacity for phagocytosis would greatly facilitate the capture of prospective endosymbionts. Extant mesophilic archaea, such as *Methanosarcinales* or *Halobacteria*, clearly have acquired numerous genes via HGT [84,98,100,101]. Moreover, the latest comparative genomic results suggest that massive acquisition of bacterial genes underlay the emergence of most if not all major archaeal phyla [79]. Conceivably, in the archaeal ancestor of eukaryotes, this gene gaining capacity was enhanced by the primitive phagocytosis, through transient engulfment of other archaea and bacteria. This ‘protophagocytic’ lifestyle would probably cause acquisition of genes from diverse bacterial sources, not the proto-mitochondrial endosymbiont alone, which could in part account for the weakness of the *α*-proteobacterial signal among the eukaryotic genes of apparent bacterial descent [159,160].

The results of phylogenomic analysis outlined above strongly suggest that the ancestor of eukaryotes was a deep branch within the TACK superphylum [81,92], possibly a distinct phylum, in addition to the currently recognized Crenarchaeota, Thaumarchaeota, Korarchaeota, Aigarchaeota, Bathyarchaeota and (possibly) Geoarchaeota (figure 1). Given that evolutionary reconstructions indicate that evolution of most of the major groups of archaea was dominated by genome reduction and streamlining [84,90], the ancestor of eukaryotes could have been a highly complex ancient archaeon. Therefore, it appears plausible that a still unidentified group of extant archaea within the TACK superphylum is a streamlined descendant of the eukaryotic ancestor (figure 1). The rapidly progressing metagenomics and especially single-cell genomic sequencing clearly have the potential to uncover this elusive ancestral group in the case that its archaeal descendants indeed have survived to this day.

If the archaeal ancestor of eukaryotes (the host of the proto-mitochondrial endosymbiont) was a complex organism with some signature features of eukaryotic cells, such as the cytoskeleton, the question emerges whether this ancestral form was an archaeon or a primitive, amitochondrial eukaryote, i.e. an archezoan. The answer hinges on the definition or rather the salient features that ‘make an organism a eukaryote’. The cytoskeleton, membrane remodelling systems such as ESCRT-III, the Ub system and RNAi certainly are eukaryotic signatures. Yet, the defining traits of eukaryotes appear to be the large size of the eukaryotic cells coupled with the presence of the elaborate endomembrane system which includes the nucleus endowed with nuclear pores and the splicing machinery that is linked to the nucleocytoplasmic trafficking. Quantitative argument has been developed that the eukaryotic cellular organization is unsustainable without multiple energy-converting organelles, such as mitochondria [1]. Furthermore, coherent scenarios have been proposed for the origin of the endomembranes, the nucleus and the spliceosome-mediated splicing in the wake of endosymbiosis [36,37,47]. The combination of these findings and inferences strongly suggests that the host of the mitochondrial endosymbiont was an archaeon although perhaps a highly complex one.

## 6. Conclusion

Four groups of recent observations increase the plausibility of the symbiogenetic scenario for the origin of eukaryotes. The first line of evidence comes from the reconstructions of archaeal genome evolution which imply complex ancestral forms, with the subsequent evolution in most lineages dominated by gene loss. The related and perhaps most important clues come from the observations on the archaeal eukaryome that is scattered among diverse extant archaea. The putative complex archaeal ancestor of eukaryotes could have encoded most if not all components of the eukaryome within the same genome, possibly endowing this ancestral archaeon with certain eukaryote-like functionalities such as the ability to efficiently engulf other cells (a primitive version of phagocytosis). The third line of evidence consists of the increasingly confident demonstrations of the origin of the core eukaryotic genes from within the archaea, or more specifically, from a deeply branching group within the TACK superphylum. Given that all known extant members of this superphylum are typical archaea and not archezoa, these findings appear to favour an archaeal host for the proto-mitochondrial endosymbiont. Finally, the indications that massive acquisition of bacterial genes most probably triggered the emergence of the major groups of archaea put the origin of eukaryotes into a more general evolutionary context. These discoveries make the origin of eukaryotes appear less dramatically different from the origin of other groups of organisms than is generally perceived. Horizontal transfer of numerous genes appeared to have been central in each case. The key difference is that in eukaryotes the source of the foreign genes, i.e. the endosymbiont, survived as an organelle, precipitating the radical restructuring of the cell. Given the likely origin of eukaryotes from within the archaeal diversity and the observations on the dispersed eukaryome, there seems to be high promise of new evolutionary insights coming from metagenomics and single-cell genomics. The discovery of archaeal descendants of the elusive host of the mitochondrial endosymbiont cannot be ruled out.

## Addendum

Shortly after this manuscript was submitted, a game-changing discovery bearing on the archaeal ancestry of eukaryotes has been published [161,162]. Deep metagenomic sequencing uncovered a remarkable group of archaea from marine sludge that combined the two key properties expected of the eukaryotic ancestor. First, one of these novel organisms, tentatively classified as a new phylum Lokiarchaeota (already affectionately known as Loki), represents a sister group to eukaryotes, and the Loki–eukaryote branch is confidently lodged deep within the TACK superphylum. Second, the genome of Loki recapitulates with an uncanny precision the reconstructed gene repertoire of the putative archaeal ancestor of eukaryotes that is outlined above. In particular, Loki encode crenactins, homologues of eukaryotic gelsolins, the ESCRT-III complex, an expanded family of small Ras-like GTPases and the complete ubiquitin system. This gene repertoire translates into a confident prediction of a complex cytoskeleton and membrane remodelling systems and is compatible with a rudimentary phagocytic capability that has been predicted for the archaeal ancestor of eukaryotes. Further exploration of the genomes and hopefully the actual biology of the Loki are likely to dramatically enhance our understanding of eukaryogenesis.
